# Association between smartphone use, sleep deprivation, and self‐rated health outcomes: A retrospective observational survey in rural Japan

**DOI:** 10.1002/jgf2.70024

**Published:** 2025-04-29

**Authors:** Yoshio Hisata, Sakiya Nishida, Mamoru Urayama, Hiroshi Sekine, Takashi Sugioka, Risa Hirata, Naoko E. Katsuki, Masaki Tago, Yuki Ueda, Masaki Amenomori, Katsumi Higashino, Yoshio Naya

**Affiliations:** ^1^ Department of Internal Medicine Nagahama City Kohoku Hospital Shiga Japan; ^2^ Department of General Medicine Saga University Hospital Saga Japan; ^3^ Department of Family Medicine Shiga Center for Family Medicine Shiga Japan; ^4^ Department of General Medicine Saga City Fuji‐Yamato Spa. Hospital Saga Japan; ^5^ Department of Internal Medicine Nishiazai Clinic Shiga Japan; ^6^ Department of Pediatrics Nagahama City Kohoku Hospital Shiga Japan; ^7^ Department of Urology Nagahama City Kohoku Hospital Shiga Japan

**Keywords:** cross‐sectional study, digital communication, self‐rated health, smartphone overuse, total sleep time

## Abstract

**Background:**

Smartphone use significantly changes the lives of individuals. This study aimed to stratify sleep time by age and sex, examine the association between smartphone overuse and sleep deprivation, and measure their impact on self‐rated health (SRH) outcomes. Focusing on communication and overuse, we also considered the association of active and passive smartphone use, as well as browsing, with sleep.

**Methods:**

We conducted a questionnaire survey among residents of three towns in rural Japan to collect data on the sleeping hours, smartphone ownership rate, and smartphone usage time of residents, stratified according to sex and age. Logistic regression analysis was conducted to investigate the association among good SRH outcomes, sleep deprivation, and smartphone overuse.

**Results:**

Among 2565 respondents to our questionnaire who possessed a smartphone (ownership 69.7%), 1172 men and 1393 women were analyzed. The mean age and sleep hours were 59.1 years and 6.7 h. Sleep time was shorter in middle‐aged residents, and sex differences disappeared at younger ages. A total of 1295 participants experienced sleep deprivation. The smartphone overuse group comprised 627 participants. Good SRH outcomes were negatively associated with smartphone overuse and sleep deprivation. The group that used their smartphones for more than 1 h slept approximately 0.12–0.13 h less than the other group. Active/passive use and screen time were associated with good SRH outcomes.

**Conclusion:**

Avoiding sleep deprivation and smartphone overuse are activities that are useful for good SRH outcomes. Smartphone use with a clear purpose may not be antagonistic to good SRH outcomes.

## Graphical abstract

Our study aimed to investigate the association among good SRH outcomes, sleep deprivation, and smartphone overuse. Good SRH outcomes were negatively associated with sleep deprivation and smartphone overuse. The group that used their smartphones for more than 1 h slept approximately 0.12–0.13 h less than within 1 h.

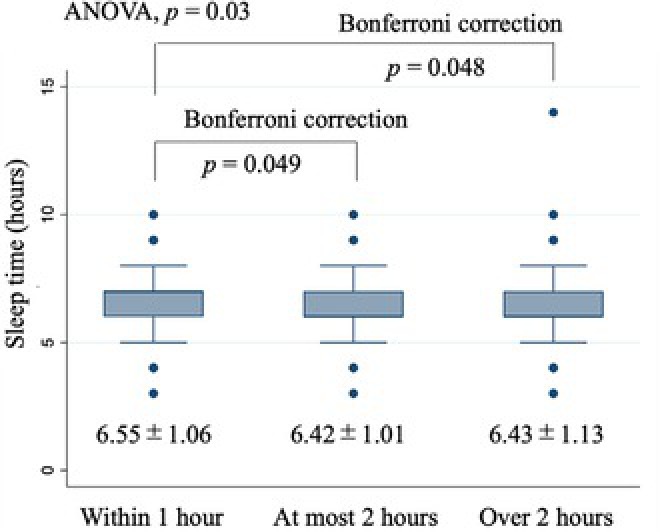

## 1 INTRODUCTION

Owning a smartphone has become common in recent years, and the smartphone ownership rate for individuals in Japan is 74.3%.^1^ Regarding the influence of smartphone usage time on sleep, using a smartphone for 2 h or more increases the time to fall asleep and shortens sleep time.^2^ The impact of sleep duration on self‐rated health (SRH) has also been examined, and several reports have been reviewed.^3^ One report showed that smartphone ownership among the elderly is associated with good SRH outcomes.^4^ However, no studies have been conducted to determine whether sleep deprivation and smartphone overuse affect SRH.

Communication through smartphones can be classified as active or passive. During active smartphone use, receiving targeted, composed communication from strong ties was associated with improvements in well‐being while viewing the wide‐audience broadcasts of friends and receiving one‐click feedback were not.^5^ During passive smartphone use, browsing social networking services (SNSs) for a long time may be stressful,^6^ and smartphone overuse has been associated with stress.^7^ Studies have also revealed an association between active and passive smartphone use among young people during the COVID‐19 pandemic and internalizing/externalizing symptoms, emphasizing the time spent viewing smartphones and the number of times they are unlocked.^8^ To our knowledge, no studies have examined how smartphone use is associated with SRH. Primary care physicians can guide lifestyle habits to improve health outcomes during outpatient treatment. We hypothesized that it is important to provide appropriate advice regarding sleep and smartphone use, considering the impact of these two factors on health.

Therefore, this study aimed to examine the association between smartphone overuse and sleep deprivation, including active/passive use and screen time devoted to internet browsing.

## 2 METHODS

### 2.1 Research design

This was a cross‐sectional study using a self‐reported questionnaire. This study comprised an exhaustive survey employing a geographic population‐based cohort approach and was conducted by the mailing method.

### 2.2 Settings

#### 2.2.1 Study population

The target population comprised all adults living in the towns of Kinomoto, Yogo, and Nishiazai, Nagahama City, Shiga Prefecture, Japan. The questionnaires were distributed from 2021 (Kinomoto and Yogo town) to 2022 (Nishiazai town). The target area was rural, with heavy snowfall and few doctors. Due to population decline and nuclear families, the children were independent and had moved out of town. Parents were often living alone or with their spouses. In general, smartphones are useful tools for contacting family members and neighbors. Amid the coronavirus disease pandemic, unnecessary outings were irregularly restricted at the national level. Another study reported that loneliness was a health problem in the same population.^9^ An environment in which face‐to‐face interaction is not possible may affect feelings of loneliness and smartphone use.

### 2.3 Measurement items

#### 2.3.1 Sociodemographic data

Characteristics such as sex, age, education, marital status, employment status, and annual household income were measured. Married individuals were likely to report good SRH outcomes compared to their never‐married counterparts.^10^ In addition, factors such as a good working environment, higher education level,^11^ and higher income^12^ are known to be associated with good SRH outcomes.

#### 2.3.2 Exposure

Additionally, we queried residents to document their sleep duration (1–23 h), whether they owned a smartphone, and the amount of time they used it, stratified into three categories: within 1 h (low), at most 2 h (middle), and over 2 h (high) per day. Sleep deprivation was defined as a sleep time of < 7 h (1–6 h), and smartphone overuse was defined as 2 h or more (high) per day, based on the findings of a previous study.^2^ We developed measurement items for the three patterns of smartphone usage with a focus on communication. For smartphone owners, the percentage of time they spent actively trying to communicate, such as by calling, emailing, or posting on SNS, was denoted as active use, and the percentage of time spent using it passively, such as receiving and checking incoming emails or simply checking SNS, was defined as passive use, while other use, such as listening to music, watching movies, or browsing the Internet, was denoted as screen time. Participants were asked to rate the percentage of communication in 10% increments (total 100%). Further, since the answers to all three patterns of smartphone usage could be 0% or 100%, we defined answers that were always 50% or higher, which is the most frequent value, as active user, passive user, and screen time user. By categorizing these three types of users with usage rates of ≥50%, the three variables become independent variables, regardless of the usage rate of the remaining two rates. Since the categories of active, passive, or screen time use are self‐reported variables created on the basis of previous studies,^8^ therefore, reliability and validity have not been verified.

#### 2.3.3 Outcome

As the main outcome for evaluating health impacts, SRH was measured using a 5‐point scale: 1: very poor, 2 = poor, 3 = intermediate, 4 = good, and 5 = very good. SRH is an indicator of mortality.^6^ We defined a good SRH outcome as a score of 4 or more. We considered a score of 4 or higher to be a clinically significant cutoff and defined it as a good SRH outcome because the mean score in previous studies examining SRH and smartphone use was 3.27 points.^4^ SRH has been reported as a predictor of mortality in several papers cited in a systematic review.^13^ We have presented a conceptual diagram in Figure S1 showing the hypothesis about factors and outcomes affecting sleep deprivation, cell phone usage, and SRH.

#### 2.3.4 Covariates

The other questions in our survey related to exercise habits,^14^ obesity,^15^ and loneliness,^9^ which were expected to be confounded by smartphone use and sleep duration. Exercise habits and obesity were measured separately because they were assumed to influence sleep duration, smartphone use, and SRH. The UCLA short version was used as the loneliness scale.^16^ A score of 1–9 was calculated, and a score of 4 or higher was defined as loneliness and used in the analysis.

### 2.4 Statistical analysis

We excluded participants who had been declined, had missing data, or did not have a smartphone. Sleep duration and smartphone use duration were analyzed according to sex and age. Using analysis of variance (ANOVA), we compared sleep time and three levels of smartphone use (low, medium, and high), and stratified sleep time and smartphone use by sex and age. A significant difference was confirmed; the Bonferroni method was performed,^17^ and the significance level was set at 0.05/3 = 0.016.

We conducted a univariate analysis to examine the association between good SRH outcomes and the measured factors using the chi‐square test. Finally, a multivariate analysis was conducted to examine the association between good SRH outcomes, sleep deprivation, and smartphone overuse (including active/passive use and screen time devoted to internet browsing). Using this model, the predictive margin of the average probability of good SRH by age and smartphone usage duration was calculated, and interaction effects were examined. Regarding confounding factors, the participants were divided into two groups based on whether they attended university, were married, employed, or had an income of 3.01 million yen or more. The STATA/BE (version 17.0) software was used.

### 2.5 Ethical considerations

Individuals were not identified in the data obtained via the survey, and informed consent was obtained upon distribution of the questionnaire, with a clear statement that respondents were free to respond or withdraw their responses. Individuals who required nursing care, those with physical or mental illnesses or disabilities, and those of foreign nationality were required to obtain the assistance of family members, acquaintances, or caregivers who could understand the situation of the respondent, the content of the text, and answer the questionnaire. We asked the participants to cooperate and answer the questions in our survey. This study was conducted with the approval of the Ethics Committee of Nagahama City Kohoku Hospital (approval no. 3 in 2020 and no. 1 in 2021).

## 3 RESULTS

**FIGURE 1:**
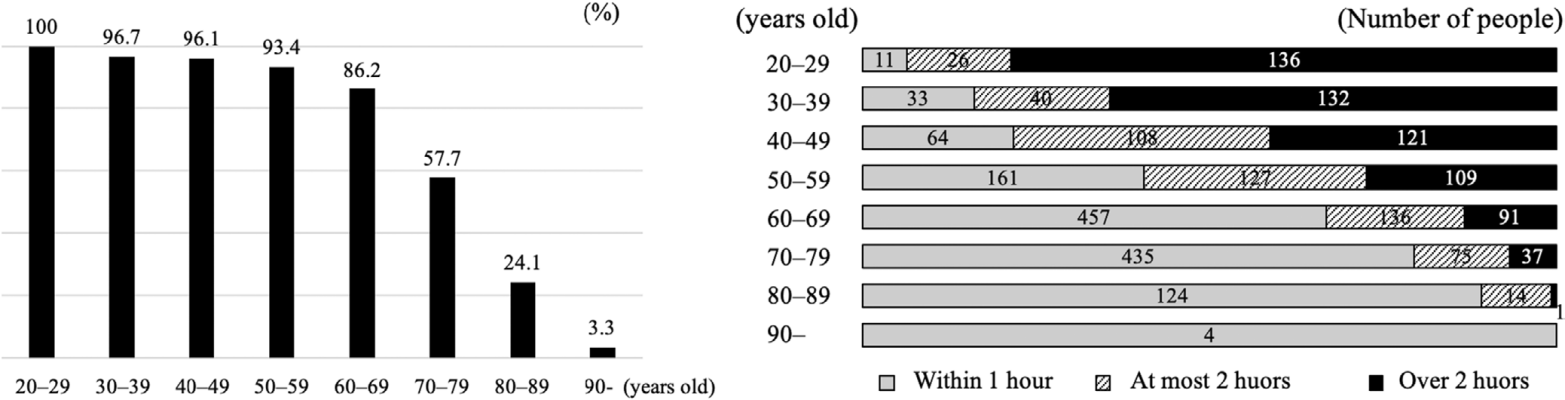
Smartphone ownership rate (left panel) and smartphone usage time distribution by age group (right panel).

Questionnaires were mailed to 11,223 individuals, and among the 3680 people who responded to it, the responses from 2565 individuals who owned smartphones were included in this study and analyzed, with a smartphone ownership rate of 69.7%. The response rate was 32.7%. The inclusion criteria are shown in Table S1. The cohort characteristics (Table 1) were as follows: 1172 men and 1393 women; mean age, 59.1 years. The mean sleep time was 6.51 h, and this is depicted by a histogram in Figure S2. Sleep deprivation (1–6 h) was assessed in 1295 participants. The smartphone usage time per day was high (over 2 h) for 627 individuals. Smartphone use time was longer in the younger age group (Figure 1, right panel). Regarding the proportion of smartphone usage pattern, 22.6% was active, 35.1% was passive, and 42.3% was screen time devoted to internet use. The good SRH outcome group (over four points) included 1835 individuals.

**TABLE 1:** Characteristics of the respondents *N* = 2565.

	Number of respondents (SD)	Percentage
Sex		
Male	1172	45.7
Female	1393	54.3
Age		
Mean	59.1 (16.3)	
Age group		
20–29	176	6.9
30–39	206	8.0
40–49	298	11.6
50–59	402	15.7
60–69	703	27.4
70–79	583	22.7
80–89	180	7.0
90–99	16	0.6
100–	1	0.04
Final education		
Up to highschool	1394	54.3
University graduate	1116	43.5
Marital state		
Get married^a^	1995	77.7
Not married	479	18.6
Employment		
Working^b^	1839	71.6
Not working/Retired	675	26.3
Household income		
<300 million yen	927	36.1
Over 301 million yen	1458	56.8
Sleep time (1–23 h)		
Mean	6.51 (1.09)	
Sleep deviation (1–6 h)	1295	50.4
Smartphone use per day		
Low (within an hour)	1289	50.2
Medium (a maximum of 2 h)	526	20.5
High (over 2 h)	627	24.4
How to use smartphone		
Active use (phone, posting mail/SNS)	22.6%	
Active user (using rate ≧50%)	294	11.4
Passive use (received mail, viewing SNS)	35.1%	
Passive user (using rate ≧50%)	677	26.3
Screen time use (music, movies, and internet)	42.3%	
Screen time user (using rate ≧50%)	955	37.2
Exercise habi		
Yes	1324	51.6
Overweight/Obesity		
Yes	673	26.2
Loneliness		
UCLA loneliness mean score (1–9 points)	4.12 (1.43)	
Loneliness (4 point or more)	1348	52.5
Self‐rated health (SRH)		
Mean score (1–5 points)	3.90 (0.99)	
Good SRH group (4 points or more)	1835	71.5

Abbreviations: SNS, social networking service; UCLA, University of California, Los Angeles.

^a^ Include bereavement.

^b^ Include housework and student.

**FIGURE 2:**
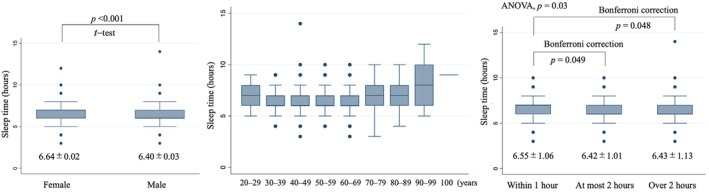
Sleep time difference by sex (left panel), age group (middle panel), and smartphone usage time (right panel).

The differences in sleep duration by sex, age group, and smartphone use time are presented in Figure 2. The sleep duration of women was shorter than that of men (men vs. women, 6.59 vs. 6.83 h, *p* < 0.001, *t*‐test) (Figure 2, left panel). Sleep time was shorter in middle age, and sex differences disappeared at younger ages (Figure 2, middle panel and Table S2).

The sleep time difference and influence of smartphone use are shown in Figure 2, right panel. The group who used their smartphone for more than 1 h slept for approximately 0.12–0.13 h less than the other respondents (*p* = 0.03, by ANOVA). A statistically significant reduction in sleep time was not confirmed when the smartphone usage time exceeded 1 h by Bonferroni correction (set at 0.016); however, for a smartphone usage of 1 h and at most 2 h, the corresponding sleep time was 6.55 versus 6.42 h, respectively (*p* = 0.049, by Bonferroni correction), while a smartphone use within 1 h and over 2 h corresponded to a sleep time of 6.55 versus 6.43 h, respectively (*p* = 0.048, by Bonferroni correction). These findings were not significant by age group, and age‐stratified data are shown in Table S3.

The results of the univariate and multivariate analyses are shown in Table 2. Without adjusting for confounding factors, a significant association was observed between good SRH outcomes and sleep deprivation (odds ratio [OR] 0.76, 95% confidence interval [CI] 0.64–0.91). Effect sizes are shown in Table S4. By contrast, no significant association was observed between good SRH outcome and smartphone use. After adjusting for confounding factors, a significantly negative association was revealed between good SRH outcomes and sleep deprivation (OR 0.68, 95% CI 0.54–0.85). Similarly, good SRH outcomes were also negatively associated with smartphone overuse (OR 0.71, 95% CI 0.53–0.95). On the other hand, a positive association was observed between good SRH outcomes and smartphone active use (OR 1.63, 95% CI 1.13–2.35), smartphone passive use (OR 1.39, 95% CI 1.005–1.92), and smartphone screen time (OR 1.47, 95% CI 1.06–2.05). In addition to the main effects, there was an interaction effect between age and smartphone overuse (Figure S3).

**TABLE 2:** Association between good self‐rated health (SRH) and measured factors.

	Univariate analysis			Multivariate analysis		
	Odds ratio	95%CI	*p*‐value	Odds ratio	95%CI	*p*‐value
Sleep deprivation	0.76	0.64–0.91	0.002	0.68	0.54–0.85	0.001
Smartphone overuse	1.12	0.91–1.38	0.26	0.71	0.53–0.95	0.02
Smartphone active use	1.24	0.92–1.68	0.14	1.63	1.13–2.35	0.008
Smartphone passive use	1.17	0.94–1.46	0.13	1.39	1.005–1.92	0.046
Smartphone screen time use	0.94	0.77–1.16	0.6	1.47	1.06–2.05	0.02
Male	0.86	0.72–1.02	0.08	0.92	0.74–1.16	0.52
Over 65 years old	0.66	0.54–0.80	<0.001	0.98	0.97–0.99	0.002
No exercise habit	0.48	0.40–0.57	<0.001	0.56	0.45–0.70	<0.001
Obesity	0.79	0.67–0.92	0.003	0.68	0.53–0.87	0.002
Loneliness	0.45	0.39–0.52	<0.001	0.52	0.41–0.65	<0.001
University graduate	1.50	1.25–1.80	<0.001	1.02	0.81–1.29	0.81
Unmarried	0.80	0.63–1.01	0.057	0.96	0.68–1.36	0.85
Non‐working	0.50	0.42–0.61	<0.001	0.54	0.40–0.72	<0.001
High income	1.57	1.30–1.89	<0.001	1.11	0.85–1.43	0.41

*Note*: Good SRH is defined in case the score is 4 points or more. Sleep deprivation is defined as 1–6 h of sleep time. Smartphone overuse is defined as using for over 2 h. Smartphone active use, passive use, and screentime are each defined as a usage rate of 50% or more.

Abbreviations: CI, confidence interval; SRH, self‐rated health.

## 4 DISCUSSION

This was a fact‐finding survey on sleep time, smartphone ownership rate, and smartphone usage time for all adults in a rural Japanese area. Japan has the largest number of older individuals globally^18^; therefore, this study could provide useful basic information on sleep and smartphone use in an aged society. Particularly, depopulated areas are heavily affected by the declining birthrate and aging population and are ahead of future Japanese demographic trends. Therefore, the present report also has value as a descriptive study. On the other hand, Japan is known to have a high sex gap^19^, ^20^ compared to other countries. Describing sex differences in sleep duration is also important in considering the elimination of the sex gap. We discuss the following three themes: sleep time by sex and age, smartphone use, and the relationship between these and health.

### 4.1 Regarding sleep time by sex and age

The sleep time of women was shorter than that of men. These results are similar to those of previous studies.^21^ In particular, working women slept for approximately 20 min less than working men in Japan,^22^ although almost no differences were noted in other countries. Women still bear the heavy burden of housework and childcare in Japan. This issue needs improvement owing to sex disparity. The sex gap in the Japanese population and its world ranking during the survey period were 0.656 in 2021 (120th/156th place) and 0.650 in −2022 (116th/146th place).^19^, ^20^ In the present study, sleep time was shorter in middle‐aged study participants, and sex differences disappeared at younger ages. Similar results were reported in Korea.^23^ The article suggested that the shorter sleep time observed in middle‐aged women was improved by socioeconomic status and recent changes in familial expectations for women more than in the past. The percentage of people who believe that men should be masculine and women should be feminine is decreasing among the younger generations.^24^ The differences in sleep duration between men and women may be corrected in a genderless society.

Sleep time decreased with age, ranging from 20 to 60 year olds. These results are similar to those of previous studies.^21^ The sleep duration for individuals aged 15–64 years in Japan was approximately 442 min (7 h 22 min) in 2016, which is at the bottom of the OECD.^25^ In the Zepp Health Corporation 2021 World Sleep White Paper, sleep time was 404 min (6 h 44 min), ranking second worst after Indonesia.^26^ In our study, the mean sleep duration per day was 390 min (6 h 30 min). Recently, the number of sleeping hours has decreased in Japan. Ensuring sufficient sleep duration is important.^27^ Among individuals in their 60s and older, the older they are, the longer they sleep. This is because older individuals have more free time after retirement and more time in bed as their bodies deteriorate and require nursing care. Data on individuals aged 65 and older were not included in the OECD review; therefore, this was a valuable result of a fact‐finding survey. A previous study reported that the mortality rate increased with a sleep time of more than 8 h.^28^ For older individuals, sleeping longer may not be beneficial.

### 4.2 Regarding smartphone ownership and its influence on sleep hours

In our study, smartphone ownership was 68.6%, lower than the 74.3% reported in other domestic studies.^1^ This was because the target population included more older individuals. The ownership rate and usage time of smartphones are naturally higher among younger individuals. As mentioned in the Introduction section, browsing SNS for a long time can be stressful,^6^ and smartphone overuse is associated with stress.^7^ Smartphone usage time within 1 h may reduce >0.1 h of sleep duration; however, this was not statistically significant. These results differed from those of a previous study.^2^ This was because smartphone use before sleep had an effect. By limiting smartphone use to approximately 1 h, one may lengthen sleep hours on days when sufficient sleep is required. Furthermore, limiting usage before sleep may be effective in ensuring adequate sleep.

### 4.3 Regarding the association between health, sleep deprivation, smartphone overuse, and pattern of use

This study revealed a significant negative association between good SRH outcomes and sleep deprivation. Similarly, good SRH outcomes were negatively associated with smartphone overuse. Regarding sleep and health, it has been reported that health‐related quality of life was reduced when the amount of sleep was <7 h.^27^ The results of the present study are in line with the previous report. Obtaining extra sleep is useful for improving health. Regarding smartphone use, it has been reported that the good SRH outcome of an older individual was associated with smartphone ownership, smartphone usage ability, and use of smartphones to learn or search for health information.^4^ In our study, smartphone overuse was positively associated with good SRH outcomes in univariate analysis. However, multivariate analysis showed that smartphone overuse had a negative tendency in the group with smartphones. This was because the study population included young individuals with smartphone addictions. A systematic review reported that the health outcomes associated with smartphone addiction in adults include depression, anxiety disorders, psychiatric symptoms, brain white matter changes and defects, musculoskeletal problems, carpal tunnel syndrome, traffic accidents, sedentary behavior, anxiety regarding coronavirus disease, increased headache duration, decreased sleep quality, reduced daytime physical activity, functional impairment, social anxiety, mental health issues, and adult attention deficit hyperactivity disorder.^29^ Appropriate use of smartphones is important, and when sleep time is considered, using a smartphone for <2 h is reasonable. Active/passive smartphone use and screen time devoted to internet use were associated with good SRH outcomes. These results were the opposite of what we would expect from excessive smartphone use. In the present study, smartphone usage activity was defined as having a usage rate > 50% for any one of the three smartphone usage patterns. These results suggest that purposeful smartphone use may not be harmful even if we use it for a long time.

## 5 LIMITATIONS

This study has a few limitations. First, this was a cross‐sectional study, which could not establish a cause‐and‐effect relationship or analyze the data over time. Therefore, longitudinal studies are warranted. Second, several factors associated with SRH were not considered. In particular, studies delving into the hypotheses regarding potential mechanisms are important. We classified three aspects of the evaluation: individual personality traits, sleep duration, and smartphone use. Individual personality traits and self‐esteem may influence SRH. Sleep time classification and quality could not be evaluated. Whether or not a person takes a nap, wake‐up time, or bedtime may be related to SRH. Moreover, whether respondents use their smartphones before going to bed and the usage conditions before going to bed can affect sleep and SRH. We could not distinguish between smartphone addiction and excessive use. We were also unable to examine the factors related to excessive smartphone use. We defined excessive smartphone use as ≥2 h based on reference literature. We also verified practical advice regarding smartphone usage time in daily life and set the limits at <1 h and 1–2 h, which are more specific based on clinical significance. Given the recent diversification of smartphone functions and length of usage time, continuous data measurement is desirable. Furthermore, the question on communication did not strictly distinguish between online and digital communication. In addition, the proportion of smartphone passive/active use in this study was self‐reported, and its reliability and validity are unclear and may not reflect the actual objective proportion of use. For example, the work performed by Marin‐Dragu et al.,^8^ active use was defined as the frequency of checking smartphones, and passive use was defined as the amount of time spent on the device; however, active use requires time to type emails and documents, whereas passive use only involves browsing. Therefore, when comparing time alone, active use is likely to be longer. The development and validation of more specific measurement methods is desirable. The respondents whose active communication was low, especially young individuals, appeared to be spending their time communicating through social games, which may not reflect the actual communication rate. This mechanism may explain the results of a previous study.^15^

## 6 CONCLUSION

Avoiding sleep deprivation and smartphone overuse are useful activities for good SRH outcomes, and it is recommended to get a minimum of 7 h of sleep and use the smartphone for a maximum of 2 h, while on days of sufficient sleep, the usage time may be restricted to within 1 h. Regarding the quality of smartphone usage, we could not find an association between active/passive use or screen time devoted to internet use and good SRH outcomes.

## FUNDING INFORMATION

The author (Yoshio Hisata) received the 2021 JA Mutual Aid Federation‐commissioned research project and the 34th regional healthcare research grant for the KYANS study.

## CONFLICT OF INTEREST STATEMENT

The authors have stated explicitly that there are no conflicts of interest in connection with this article.

## ETHICS STATEMENT

Ethics approval statement: This study was approved by the Ethics Committee of Nagahama City Kohoku Hospital (Approval No. 3 in FY 2019, No. 1 in FY 2021, and No. 1 in FY 2023).

Patient consent statement: Consent was obtained after clearly stating that the respondents were free to respond or withdraw their responses.

Clinical trial registration: None.

## Supporting information


Figures S1–S3

Tables S1–S4

## Data Availability

The data that support the findings of this study are available from the corresponding author, Yoshio Hisata, upon reasonable request.
