# Supplementary figures and images for: Association between smartphone use, sleep deprivation, and self‐rated health outcomes: A retrospective observational survey in rural Japan
Source: J Gen Fam Med. 2025 Apr 29;26(5):408–15. doi: 10.1002/jgf2.70024 (PMC12404160; doi:10.1002/jgf2.70024)

## Slide 1
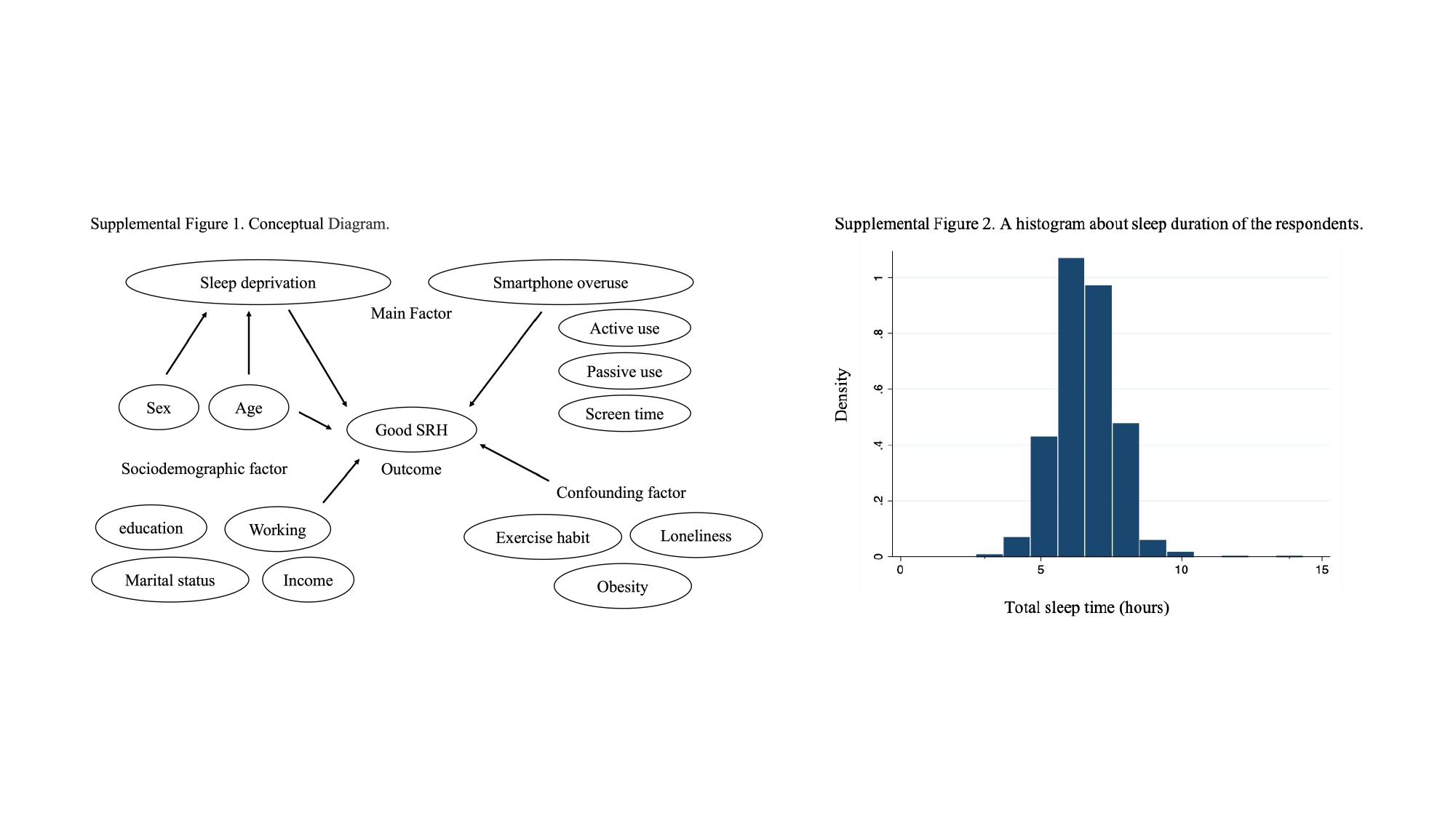

## Slide 2
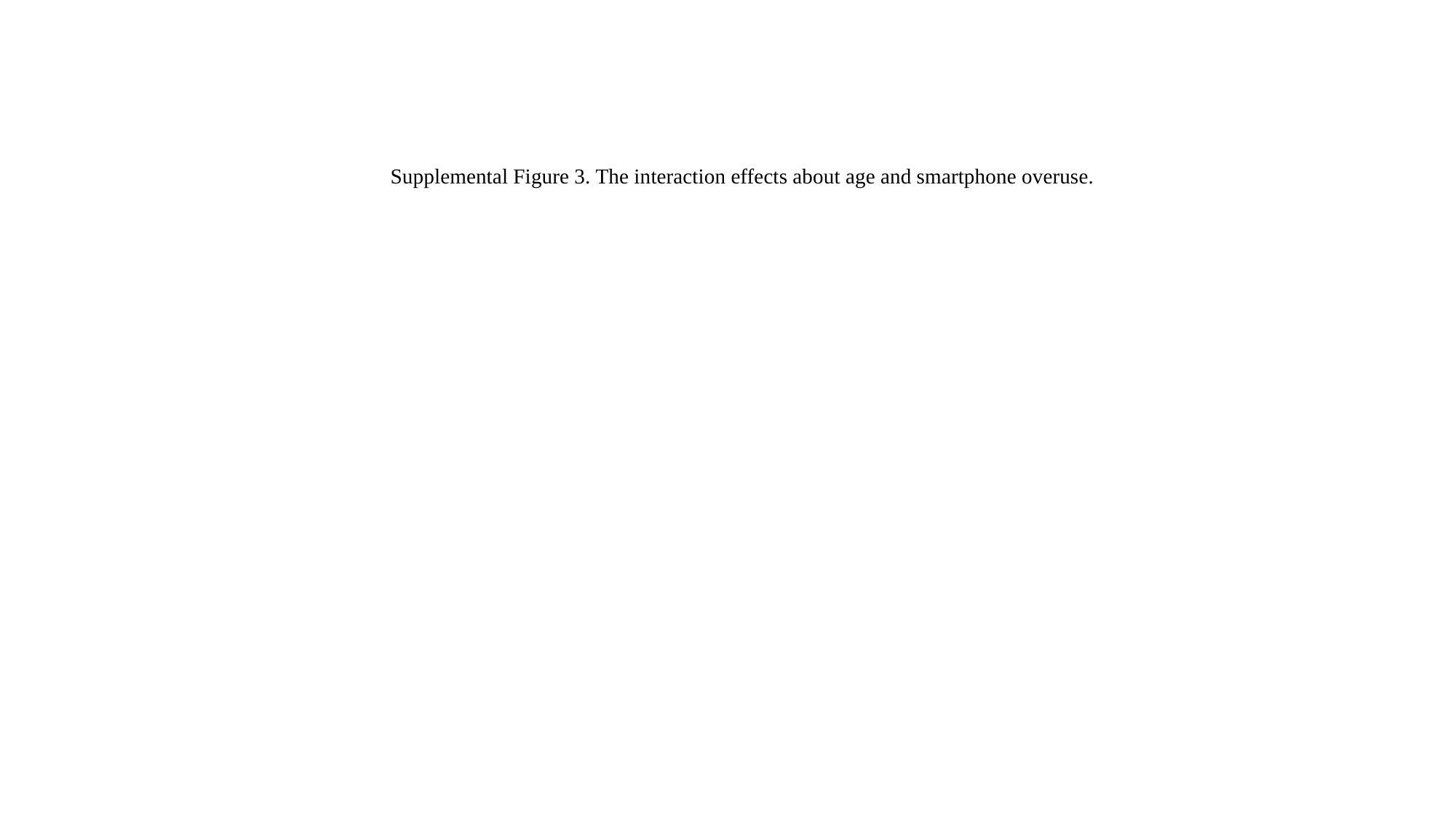

Supplemental Figure 3. The interaction effects about age and smartphone overuse.

Supplement: Supplementary file 1 — Figures S1–S3 [file JGF2-26-408-s002.pptx]
